# Comparison of the Effects of Full Median Sternotomy *vs.* Mini-Incision on Postoperative Pain in Cardiac Surgery: A Meta-Analysis

**DOI:** 10.21470/1678-9741-2023-0154

**Published:** 2024-05-13

**Authors:** Antonio de Jesus Chaves Junior, Paula Stelitano Avelino, Jackson Brandão Lopes

**Affiliations:** 1 Faculdade de Medicina da Bahia, Universidade Federal da Bahia (FMB/UFBA), Salvador, Bahia, Brazil; 2 Faculdade de Medicina, Universidade Salvador (UNIFACS), Salvador, Bahia, Brazil; 3 Department of Anesthesiology and Surgery, Faculdade de Medicina da Bahia, Universidade Federal da Bahia (FMB/UFBA), Salvador, Bahia, Brazil

**Keywords:** Cardiac Surgical Procedures, Coronary Artery Bypass, Postoperative Pain, Aortic Valve, Mitral Valve

## Abstract

**Introduction:**

It is not yet clear whether cardiac surgery by mini-incision (minimally invasive cardiac surgery [MICS]) is overall less painful than the conventional approach by full sternotomy (FS). A meta-analysis is necessary to investigate polled results on this topic.

**Methods:**

PubMed®/MEDLINE, Cochrane CENTRAL, Latin American and Caribbean Health Sciences Literature (or LILACS), and Scientific Electronic Library Online (or SciELO) were searched for all clinical trials, reported until 2022, comparing FS with MICS in coronary artery bypass grafting (CABG), mitral valve surgery (MVS), and aortic valve replacement (AVR), and postoperative pain outcome was analyzed. Main summary measures were the method of standardized mean differences (SMD) with a 95% confidence interval (CI) and P-values (considered statistically significant when < 0.05).

**Results:**

In AVR, the general estimate of postoperative pain effect favored MICS (SMD 0.87 [95% CI 0.04 to 1.71], P=0.04). However, in the sensitivity analysis, there was no difference between the groups (SMD 0.70 [95% CI -0.69 to 2.09], P=0.32). For MVS, it was not possible to perform a meta-analysis with the included studies, because they had different methodologies. In CABG, the general estimate of the effect of postoperative pain did not favor any of the approaches (SMD -0.40 [95% CI -1.07 to 0.26], P=0.23), which was confirmed by sensitivity analysis (SMD -0.02 [95% CI -0.71 to 0.67], P=0.95).

**Conclusion:**

MICS was not globally less painful than the FS approach. It seems that postoperative pain is more related to the degree of tissue retraction than to the size of the incision.

## INTRODUCTION

**Table:** 

Abbreviations, Acronyms & Symbols				
ASA	= American Society of Anesthesiologists		MVRr	= Mitral valve repair
AVR	= Aortic valve replacement		MVRt	= Mitral valve replacement
CABG	= Coronary artery bypass grafting		MVS	= Mitral valve surgery
CI	= Confidence interval		NRCT	= Non-randomized clinical trial
CPB	= Cardiopulmonary bypass		NRS	= Numerical rating scale
EuroSCORE	= European System for Cardiac Operative Risk Evaluation		NS	= Not specified
FS	= Full sternotomy		NSAID	= Non-steroidal anti-inflammatory drugs
LILACS	= Latin American and Caribbean Health Sciences Literature		PBRS	= Pain behavior rating scale
LITA	= Left internal thoracic artery		QRCT	= Quasi-randomized clinical trial
MD	= Mean difference		RCT	= Randomized clinical trial
MERSS	= Metabolic and endocrine response to surgical stress		SciELO	= Scientific Electronic Library Online
MICS	= Minimally invasive cardiac surgery		SD	= Standard deviation
MIDCABG	= Minimally invasive direct coronary artery bypass grafting		SMD	= Standardized mean difference
MIDCAB-conv	= MIDCAB with dissection of the LITA under direct vision		VAS	= Visual analog scale
MIDCABG-endo	= MIDCABG with endoscopic dissection of the LITA		VRS	= Verbal rating scale

Postoperative pain contributes directly to the intensity of the metabolic and endocrine response to surgical stress (MERSS). The effective control of algesia - either through the appropriate choice of the surgical and anesthetic technique or by the administration of analgesics in the pre and/or postoperative period - aims to reduce the mechanism of the nociceptive pain, which contributes to minimizing the impact on organs and system functions, resulting in less morbidity^[1]^. Less painful surgery can improve MERSS and can be helpful in the management of postoperative pain, requiring smaller doses of opioids and their undesirable adverse effects: sedation, respiratory depression, delirium, nausea, vomiting, paralytic ileus, and tolerance^[2]^.

Nowadays, a structural heart defect can be managed in many ways with many approaches already available and with postoperative results reported^[3]^. Among the possibilities, in addition to full median sternotomy, we can list partial sternotomy, mini-thoracotomy (including video-assisted and/or robotic surgery), and percutaneous and hybrid procedures^[3]^.

It is expected that smaller incisions can be less painful than full sternotomy (FS), but, considering the postoperative pain evaluation scores, it is still unclear whether cardiac surgery by mini-incision (minimally invasive cardiac surgery [MICS]) is superior to the conventional technique, even though one of the first MICS dates back to around 1995^[4]^. Data from previous studies are not conclusive, so efforts should be made to review the main evidence in this field to establish a greater clarification and contribute to the decision-making process of the teams involved with invasive heart procedures. Thus, this study aimed to compare the intensity of postoperative pain, measured at least in one moment over the first seven days, between FS and mini-incision in myocardial revascularization surgeries, mitral valve surgery (MVS), and aortic valve replacement (AVR). We analyzed postoperative pain as a primary outcome. The difference in the demand for analgesics between the groups in the postoperative days was analyzed as a secondary outcome.

## METHODS

This work is a systematic review of the literature with meta-analysis. The articles were searched in the MEDLINE/PubMed®, Cochrane CENTRAL, Latin American and Caribbean Health Sciences Literature (or LILACS), and Scientific Electronic Library Online (or SciELO) databases reported until 2022, following the criteria of the Preferred Reporting Items for Systematic Review and Meta-analysis (or PRISMA) databases. This study was registered at PROSPERO as CRD42021252248.

### Eligibility Criteria

In the selection, we included studies according to these criteria: randomized clinical trials (RCT) or non-randomized clinical trials (NRCT) comparing the FS technique with MICS on MVS, AVR, or coronary artery bypass grafting (CABG); clinical trials investigating the outcome of postoperative pain at least over one moment in the first few days, comparing the two approaches; papers published in any year; and publications in English, Portuguese, and Spanish. Studies conducted with pediatric patients, and studies comparing exclusively percutaneous coronary intervention or transcatheter valve repair with the standard cardiac surgical approach were excluded.

### Search Strategy

The descriptors used in the databases were: [Sternum/Surgery], [Sternotomy], [Minimally Invasive Surgical Procedures], [Thoracotomy], [Thoracoscopy], [Thoracic Surgery, Video-Assisted], [Postoperative Period], [Postoperative Complications], [Pain, Postoperative], and [Pain]. [AND] and [OR] were used as Boolean operators in the search strategy.

### Selection of Studies and Data

The articles were evaluated by reading the title and abstract and included in the final selection after reading the full text by two independent reviewers, considering the match to the established inclusion criteria. We also included papers that were cited by the articles of the consulted bibliography and that fulfill the selection criteria to increase the sensitivity of the search. The divergences between the reviewers were solved by consensus or in cases of discordance, a third reviewer was called. The pain scales used in the studies were the visual analog scale (VAS) in eleven studies (79%), numerical rating scale in three studies (21%), and the verbal rating scale in two studies (14%). In one study (7%), a pain behavior rating scale (PBRS) was used. As the study that evaluated pain through the PBRS also evaluated it by VAS, we used the VAS values to perform statistical calculations. We followed this same rule for the other studies that used more than one scale and included VAS to establish a greater standardization in the calculation of meta-analysis, also considering that VAS was the most frequently used scale. In the analysis of analgesic demand, categorical and continuous variables were used to measure the effect size, as the information on higher or lower doses of analgesics used a percentage of patients who used additional analgesics and the total dose administered in milligrams.

### Evaluation of the Quality of Studies

The Jadad score^[5]^ was used to evaluate the methodological quality of the studies. The quality scale ranges from 0 to 5 points, with a score of ≤ 2 indicating a low-quality report and a ≥ 3 score indicating a high-quality report.

### Statistical Analysis

Given the variability of methods for pain assessment, including the use of different scales, we utilized the standardized mean difference (SMD) method to perform the statistical analysis, always accompanied by 95% confidence intervals (CIs). No scale presented an inverse direction, and no conversion was required. In the meta-analysis for analgesics demand, the mean difference (MD) method was used.

Some studies registered two or more levels of pain per group at different times. Therefore, to the effect of analysis, we always used the first point observation, because it is considered more clinically significant. Only in one study, we used the second observation because it was not possible to extract dispersion data from the first. In studies that did not report standard deviation for the groups, only mean and *P*-value had the standard deviation determined through the critical value of *t*, extracted from the *P*-value and degrees of freedom. With the critical value of *t*, a standard error was obtained. With the standard error value and sample size, it was possible to impute the standard deviation, as established by Cochrane Handbook for Systematic Reviews of Interventions^[6]^. In one study, the *P*-value was not accurate but indicated as statistically significant (*P*<0.05). In this situation, we adopted the conservative point of view by assuming the critical value of *t* to *P*-value = 0.499.

The I^2^ statistic was used to evaluate heterogeneity for each analysis. Once important heterogeneity (I^2^ > 50%) could be identified, we reported and explored possible causes.

The RevMan Version 5.4 (free download from https://training.cochrane.org/online-learning/core-software/revman) software was used through meta-analysis with random effects from SMD, considering the methodological variability among the studies. Meta-analyses were performed separately for each intervention: myocardial revascularization surgery (CABG), MVS, and AVR. The method with random effects was used considering the variability of the intervention (different surgical techniques) and variability in the outcome (different pain scales).

### Sensitivity Analysis

We plan to perform sensitivity analysis including only high-quality studies by the Jadad scale^[5]^. As only a small number of the studies included were considered as high quality, we performed the sensitivity analysis as a second form of analysis to measure the size of the effect, which was performed with the observations of the groups combined into a single value and subsequent meta-analysis. In this model, the combination of means at different times was obtained using a simple mean. The standard deviations could not be combined because these data were paired observations. Thus, we performed the imputation of the data from the square root of the mean of variances, divided by the number of measurements. The choice of the imputation method was made based on its similarity to the process of obtaining the combined mean.

## RESULTS

**Fig. 1:**
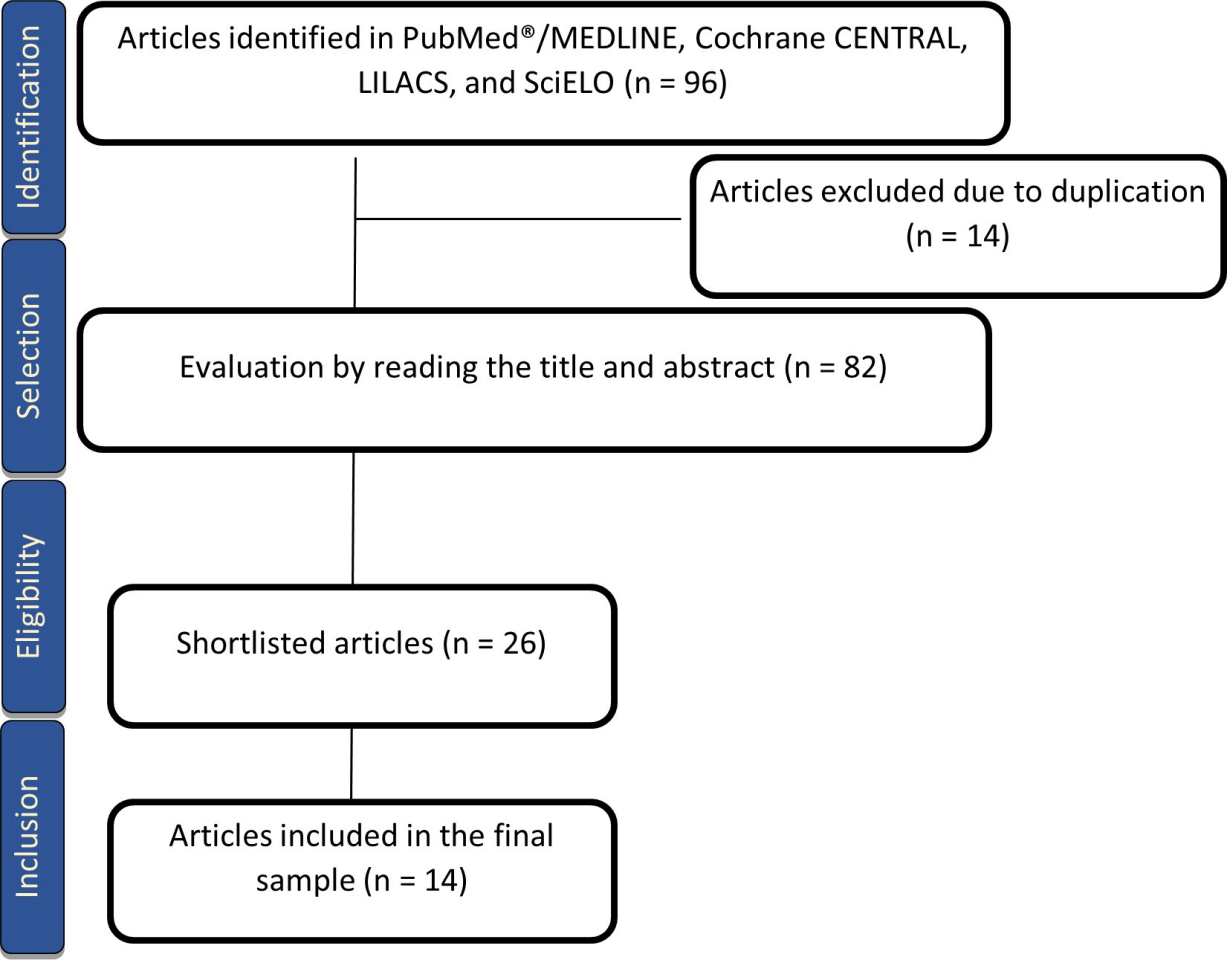
Flowchart of the article collection. LILACS=Latin American and Caribbean Health Sciences Literature; SciELO=Scientific Electronic Library Online.

A total of 96 studies were found. Of these, 14 were excluded due to duplication. After reading the title and abstract, 26 articles were selected for a full reading. Fourteen articles were selected in the final sample, including those added by reference reading (Figure 1). A total of 1,416 patients were analyzed (711 FS *vs.* 705 MICS), and these included adults from centers in Germany, Spain, Italy, Brazil, France, India, and Egypt.

From the 14 articles included in the final selection, nine were RCTs^[7-15]^, one was a quasi-randomized clinical trial (QRCT)^[16]^, and four were NRCTs^[17-20]^. The FS technique was compared to MICS: left mini-thoracotomy with dissection of the left internal thoracic artery (LITA) under direct or endoscopic vision and lower partial sternotomy to CABGs; right mini-thoracotomy to mitral valve procedures; upper partial sternotomy and right mini-thoracotomy to aortic valve procedures. Table 1 summarizes all relevant information and the assessment of the quality of the included studies. Overall, the studies showed low quality. Only two articles were considered of high quality, and 12 were considered of low quality by the Jadad scale.

**Table 1:** Study characteristics and quality assessment.

Author, year, and place	Study design	Population	Mini-incision technique	Surgical risk	Pain scale	Anesthetic regimen	Jadad scale
Ahangar AG et al. (2013), India	RCT	60 patients, eligible for AVR	Right mini-thoracotomy	ASA 1 and 2	NRS	Intraoperative: NS	2
		FS = 30				Postoperative: intravenous morphine, 3 mg, every 6 hours, in both groups	
		MICS = 30					
Aris A et al. (1999), Spain	RCT	40 patients, eligible for AVR	“J” or inverted “L” or “C” ministernotomy	5% to 10% (Parsonnet score)	VAS	Intraoperative: etomidate, fentanyl, pancuronium, and propofol in both groups	2
		FS = 20				Postoperative: continuous infusion of metamizole (dipyrone) at the rate of 4 g/12 hours while in the intensive care unit and oral acetaminophen (500 mg) and codeine (30 mg) while on the floor in both groups	
		MICS= 20					
Bonacchi M et al. (2002), Italy	RCT	80 patients, eligible for AVR	Inverted “L” or “C” ministernotomy	NS	NRS	Intraoperative: not detailed, just described as the same in both groups	2
		FS = 40				Postoperative: morphine and ketorolac-tromethamine in both groups	
		MICS = 40					
Bucerius J et al. (2002), Germany	QRCT	190 patients, eligible for CABG	MIDCAB-conv: left mini-thoracotomy	NS	VAS and VRS	Intraoperative: propofol, sufentanyl, pancuronium, bromide, and sodium thiopental in both groups	0
		FS = 93	MIDCAB-endo: left mini-thoracotomy with endoscopic LITA removal using the da Vinci surgical system			Postoperative: morphine-like analgesics upon demand in both groups	
		MICS:					
		MIDCAB-conv. = 73					
		MIDCAB-endo. = 24					
Calderon J et al. (2009), France	RCT	78 patients, eligible for AVR	Inverted “L” ministernotomy	≤ ASA 3	VAS	Intraoperative: propofol, remifentanil, and cisatracurium in both groups	2
		FS = 39				Postoperative: 1 g of paracetamol intravenously every 6 hours and patient controlled analgesia with morphine infusion during the 2 planned days of stay in the intensive care unit in both groups. Ketoprofen, 50 mg every 8 hours was infused if morphine and paracetamol were not enough	
		MICS = 39					
Rogers CA, et al. (2013), Italy	RCT	184 patients, eligible for CABG without CPB	Left mini-thoracotomy	Low and medium risk by EuroSCORE	VAS or VRS for patients unable to use VAS	Intraoperative: NS	3
		FS = 93				Postoperative: at MICS, a paravertebral block was performed through a catheter with an infusion of 0.125% bupivacaine, 5-10 mL/h, plus a bolus through the catheter and intercostal injections of bupivacaine before chest closure. In case of failure, new blocks, ketorolac/diclofenac, morphine, or ketamine were administered. There were no specifications for FS	
		MICS = 91					
Gulielmos V et al. (1999), Germany	RCT	36 patients, eligible for CABG	Left mini-thoracotomy	NS	VAS and PBRS	Intraoperative: enflurane, fentanyl, and vecuronium in both groups	1
		FS = 19				Postoperative: NS	
		MICS = 17					
Dogan S et al. (2003), Germany	RCT	40 patients, eligible for AVR	Inverted “L” ministernotomy	NS	VAS	Anesthetic regimen not detailed, just described as the same in both groups	1
		FS = 20					
		MICS = 20					
Fareed S et al. (2018), Egypt	RCT	60 patients, eligible for AVR	“J” ministernotomy	NS	VAS	Anesthetic regimen not detailed, just described as the same in both groups	1
		FS = 30					
		MICS = 30					
Speziale G et al. (2011), Italy	RCT	140 patients, eligible for MVRr	Right mini-thoracotomy	NS	VAS	Intraoperative: NS	3
		FS = 70				Postoperative: ketorolac 30 mg and indomethacin 50 mg in both groups	
		MICS = 70					
Lichtenberg A et al. (2000), Germany	NRCT	30 patients, eligible for CABG	Left mini-thoracotomy	NS	NRS	Intraoperative: etomidate, fentanyl, pancuronium bromide, and sodium thiopental	0
		FS = 15				Postoperative: NS	
		MICS = 15					
Gulielmos V et al. (1999), Germany	NRCT	122 patients, eligible for CABG	Left mini-thoracotomy	NS	VAS	Anesthetic regimen not detailed, just described as the same in both groups	0
		FS = 53					
		MICS = 69					
Guizilini S, et al. (2010), Brazil	NRCT	18 patients, eligible for CABG	Ministernotomy in the lower portion of the sternum	NS	VAS	Intraoperative: NS	0
		FS = 10				Postoperative: tramadol 100 mg four times/day in both groups	
		MICS = 8					
Walther T et al. (1999), Germany	NRCT	338 patients, eligible for CABG (160); MVRt or MVRr (58); and AVR (120)	CABG:	NS	VAS	NS	0
		CABG:	left mini-thoracotomy				
		FS = 65	MVRr or MVRt:				
		MICS = 95	right mini-thoracotomy AVR:				
		MVRr or MVRt:	ministernotomy				
		FS = 30					
		MICS = 28					
		AVR:					
		FS = 84					
		MICS = 36					

ASA=American Society of Anesthesiologists; AVR=aortic valve replacement; CABG=coronary artery bypass grafting; CPB=cardiopulmonary bypass; EuroSCORE=European System for Cardiac Operative Risk Evaluation; FS=full sternotomy; LITA=left internal thoracic artery; MICS=minimally invasive cardiac surgery; MIDCABG=minimally invasive direct coronary artery bypass grafting; MIDCAB-conv=MIDCAB with dissection of the LITA under direct vision; MIDCABG-endo=MIDCABG with endoscopic dissection of the LITA; MVRr=mitral valve repair; MVRt=mitral valve replacement; NRS=numerical rating scale; NRCT=non-randomized clinical trial; NS=not specified; PBRS=pain behavior rating scale; QRCT=quasi-randomized clinical trial; RCT=randomized clinical trial; VAS=visual analog scale; VRS=verbal rating scale

In the MVS, there was an RCT^[7]^ and a QRCT^[17]^. It was not possible to perform the meta-analysis due to the methodological differences among the studies. However, we were able to find unanimity among the authors, favoring MICS through right mini-thoracotomy, when considering the postoperative pain outcome.

**Fig. 2:**
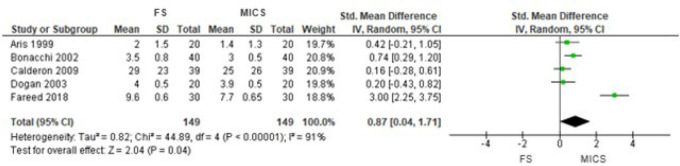
Comparison of full sternotomy (FS) vs. minimally invasive cardiac surgery (MICS) in aortic valve replacement in randomized clinical trials. Outcome 1: postoperative pain using 1st observation. CI=confidence interval; SD=standard deviation.

**Fig. 3:**
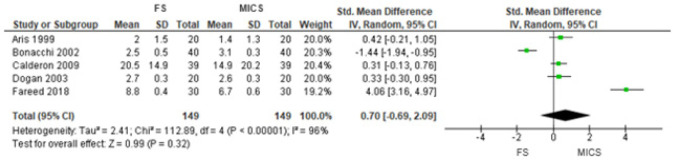
Comparison of full sternotomy (FS) vs. minimally invasive cardiac surgery (MICS) in aortic valve replacement in randomized clinical trials. Outcome 1: sensitivity analysis. CI=confidence interval; SD=standard deviation.

Considering AVR, we found a difference in the general estimation of the effect of postoperative pain in RCTs^[8-12]^, favoring MICS (SMD 0.87 [95% CI 0.04 to 1.71], *P*=0.04) (Figure 2). However, in the sensitivity analysis, using the mean of the means and the square root of the mean of variances divided by the number of measurements, there was no difference between the groups (SMD 0.70 [95% CI -0.69 to 2.09], *P*=0.32) (Figure 3). These studies compared FS *vs.* the MICS technique by upper partial sternotomy. Ahangar^[13]^ compared FS *vs.* right mini-thoracotomy in AVR and favored MICS. However, this was not included in the meta-analysis because this approach by MICS differed considerably from the others in AVR.

**Fig. 4:**
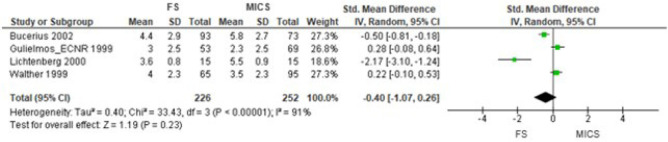
Comparison of full sternotomy (FS) vs. minimally invasive cardiac surgery (MICS) in coronary artery bypass grafting in non-randomized clinical trials. Outcome 1: postoperative pain using 1st observation. CI=confidence interval; SD=standard deviation.

**Fig. 5:**
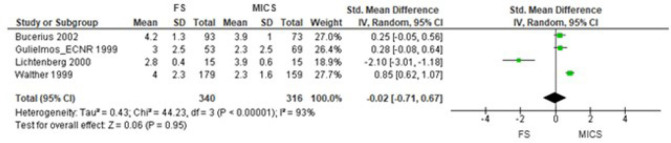
Comparison of full sternotomy (FS) vs. minimally invasive cardiac surgery (MICS) in coronary artery bypass grafting in non-randomized clinical trials. Outcome 1: sensitivity analysis. CI=confidence interval; SD=standard deviation.

**Fig. 6:**
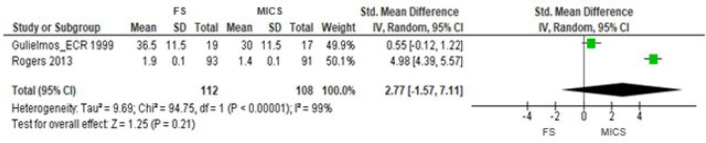
Comparison of full sternotomy (FS) vs. minimally invasive cardiac surgery (MICS) in coronary artery bypass grafting in randomized clinical trials. Outcome 1: sensitivity analysis. CI=confidence interval; SD=standard deviation.

In CABG, the general estimation of the effect of postoperative pain, performing meta-analysis with three NRCTs^[17-19]^ and one QRCT^[16]^, did not favor any of the approaches (SMD -0.40 [95% CI -1.07 to 0.26], *P*=0.23) (Figure 4). This result was also confirmed by sensitivity analysis (SMD -0.02 [95% CI -0.71 to 0.67], *P*=0.95) (Figure 5). In the meta-analysis of two RCTs^[14,15]^, the general estimate of effect using the methodology for sensitivity analysis also confirmed these results (SMD 2.77 [95% CI -1.57 to 7.11], *P*=0.21) (Figure 6). In these RCTs, we did not calculate the general effect estimate using the first observation, as one author^[15]^ did not inform the dispersion data and performing the reverse process with the *P*-value, the determination of the standard deviation was 0, making it impossible to estimate the measurement of SMDs. Bucerius et al.^[16]^ used three subgroups to compare the results, which included CABG through FS, left mini-thoracotomy CABG harvesting LITA under direct vision, and left mini-thoracotomy CABG harvesting LITA under endoscopic vision. Because a small number of patients were randomized to the harvesting of LITA under direct or endoscopic vision, it was considered a QRCT and was compared with NRCT. Despite the methodological limitation, it demonstrated that the intensity of pain was higher on the first postoperative day in MICS with LITA harvesting under direct vision and decreased until it became equal or lower concerning FS in subsequent days. It was also found that, with endoscopic harvesting of LITA, the pain was the same as FS on the first postoperative day, but decreased significantly during the follow-up, becoming less intense than FS. Only Guizilini et al.^[20]^ observed higher pain levels in the first postoperative days through FS. However, this study was not included in the meta-analysis because the MICS technique (inferior partial sternotomy) differed considerably from the other studies.

**Fig. 7:**
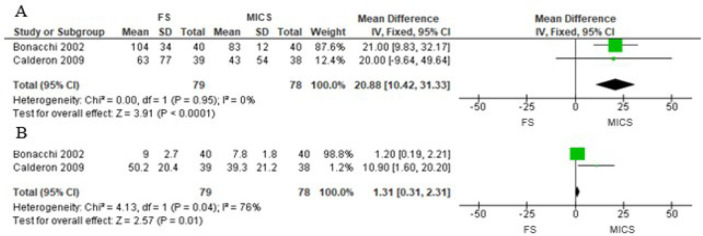
Comparison of full sternotomy (FS) vs. minimally invasive cardiac surgery (MICS) in aortic valve replacement in randomized clinical trials. Outcome 2: demand for analgesics. A) In milligrams of non-steroidal anti-inflammatory drugs, and B) in milligrams of morphine. CI=confidence interval; SD=standard deviation.

Four studies investigated the demand for analgesics in the postoperative period^[9,11,16,19]^, however, none evaluated this outcome in MVS. For CABG, Rogers et al.^[15]^, in an RCT, found that the demand for analgesics was higher in the group that underwent mini-thoracotomy and harvesting LITA under direct vision, corroborating Bucerius et al.^[16]^ findings. The latter author also observed that, with endoscopic LITA harvest, the demand for analgesics was lower compared to the FS group. This author applied two different ways of evaluation, only citing the group in which there was a greater demand and the additional percentage of analgesics received; therefore, it was not possible to perform the meta-analysis. In AVR, two RCTs^[9,11]^ were used as an estimate of effect size, the total dose of non-steroidal anti-inflammatory drugs (NSAIDs) and morphine, in milligrams, administered in the first three postoperative days. Thus, it was possible to perform the meta-analysis, and the general estimate of the effect size showed higher demand for NSAID and morphine in the FS group (Figure 7) (MD 20.88 [95% CI 10.42 to 31.43], *P*<0.0001 and MD 1.31 [95% CI 0.31 to 2.31], *P*=0.01, respectively).

## DISCUSSION

This systematic review with meta-analysis evaluated the difference in pain intensity and demand for analgesics in the postoperative period of cardiac surgery by comparing FS cardiac surgery and MICS in the subgroups that underwent CABG, MVS, and AVR. We found nine RCTs^[7-15]^, four NRCTs^[17-20]^, and one QRCT^[16]^, with 1,416 patients who received the intervention and were evaluated according to the study question.

Some limitations in the analysis of postoperative pain could be identified during the evaluation. Using the first point of pain measurement, we can recognize a reporting bias. When combining the observations of the groups and transforming them into a single value, there is a limitation due to the need to assign the dispersion measure, the standard deviation. Therefore, the choice for the first observation as the unit of analysis occurred because it was clinically more relevant and related to subsequent measurements. In addition, it can be considered simple and therefore easier to interpret. The combination of means and variances considers multiple observations and may be more accurate, so it was used for sensitivity analysis.

In general, the quality of the studies found was low. Blinding, which is often reported in controlled studies, was also a limitation identified. In this review, no author declared double blinding. In addition, some studies did not perform randomization. These were the main limitations that we found in this review, and it was what compromised the quality of the studies the most, since, using the Jadad scale^[5]^ for evaluation, four points are already lost in these two aspects.

We found important statistical heterogeneity, and the variation in the estimation of effect size among the studies was already expected due to differences in their sample sizes and generally low quality. The management of postoperative surgical analgesia differed and was also an important factor that contributed to this heterogeneity. We could see, for example, that Calderon et al.^[11]^ were more permissive in the administration of morphine, while Bonacchi et al.^[9]^ prioritized NSAIDs. Other important limitations were the relatively small size of the study samples and a substantial variety of approaches in MICS, mainly for AVR. In this surgery, to mitigate this problem, Malaisrie et al.^[21]^ call for MICS AVR standardization, so the benefits of this technique can be easier verified, which will greatly facilitate the design and implementation of future clinical studies. In addition, we found a certain variety in the study design of the selected works. To minimize these effects on the results, we performed separate analyses of randomized and non-randomized studies. Thus, we observed that, despite the difference in the study design of the works in this review, in the end, the subgroups were grouped by studies with similar designs (Figures 2 to 7), which contributed to the reduction of the effect of heterogeneity in the general scrutiny of the findings.

Performing an extensive literature search over similar studies, other reviews were retrieved that analyzed postoperative pain in specific surgical approaches, such as AVR and MVS. But no similar review was found for CABG. Two reviews, with meta-analysis for postoperative pain, found no difference between FS and MICS in AVR^[22,23]^. In MVS, two systematic reviews described that MICS had lower levels of pain in the included studies^[24,25]^.

In a meta-analysis, Kirmani et al.^[22]^ included only RCTs with patients who underwent AVR and found no difference in postoperative pain between the two groups. The studies included by this author in the analysis were also found in our search and were part of this review^[9,11,12]^. Accordingly, Lim et al.^[23]^ also found no difference in postoperative pain levels. This author included RCTs, which were also found in our search^[9-12]^, and observational studies with propensity score matches, but with separate meta-analyses. We verified higher levels of pain through FS in AVR when we used the first observation pain measurement method. This divergence with the other authors may have occurred due to the inclusion of a more recent RCT^[8]^, which was the study that found the largest difference in favor of MICS. However, in the sensitivity analysis using the mean of the means and the square root of the mean of variances, divided by the number of measurements, which provided a more global estimate of the effect, we did not find a statistically significant difference between the groups, keeping the difference in pain closer to the results of the other studies that evaluated FS *vs.* MICS in AVR.

We verified a higher demand for NSAID and morphine in AVR in the first postoperative days in the group that underwent FS.

In the MVS, the two reviews that analyzed postoperative pain did not perform a meta-analysis of this outcome^[24,25]^. In the description of the results, Modi et al.^[24]^, who selected RCTs, cohorts, and case-control studies, found a statistically significant difference in the studies selected, favoring MICS. Mariscalco et al.^[25]^, examining RCTs and observational studies, also found lower levels of postoperative pain in mitral MICS. As reported by the other authors, we also found that, in the included studies, the approach by MICS presented lower levels of postoperative pain when compared to FS. However, one limitation is that these findings are based on observational studies and a limited number of RCTs. Therefore, caution should be exercised in interpreting these data. In our review, it was not possible to perform a meta-analysis of the data on MVS studies, as they presented different methodologies.

We did not verify a difference in postoperative pain in CABG between FS and MICS. When we performed the sensitivity analysis, this characteristic was confirmed. Some authors compared postoperative pain between FS and MICS with the harvesting of LITA under direct vision. Bucerius et al.^[16]^ was the only author who compared postoperative pain between FS and MICS with the harvesting of LITA under the endoscopic vision and found that, in this case, the pain was lower in the MICS group. One study, by Guizilini et al.^[20]^, made the MICS approach through a lower partial sternotomy and reported lower pain levels in the MICS group. An interesting feature demonstrated by Trehan et al.^[26]^, a clinical trial that included 534 patients, and which to some extent corroborates the results found by Guizilini, is that when the lower partial sternotomy was compared to single left internal mammary artery graft CABG through left mini-thoracotomy, lower pain levels and lower need for analgesics were verified with a statistically significant difference after the second postoperative day, favoring partial lower sternotomy. Thus, we observed that the incision in the lower part of the sternum may be the less painful approach to single graft CABG.

A plausible justification for these findings in cardiac surgery is that postoperative pain may be more related to the degree of retraction and pressure of the retractor on the tissues than to the size of the incision. In the analysis of MICS - CABG by left mini-thoracotomy and endoscopic harvesting of LITA^[16]^ -, in which the degree of retraction and tissue pressure in these approaches is less intense than when LITA is harvested under direct vision, relative pain levels were lower. The findings of Trehan et al.^[26]^ suggest that the topography of the incision in the lateral region in the thorax may also be an important variable to predict the higher elevation of postoperative pain levels. This may be related to the injury of the intercostal nerves, which pass lower than the ribs, at the time of use of the retractors, causing nerve injury due to neuropraxia. In any case, it appears that the degree of retraction and tissue pressure is the main predictor variable of postoperative pain, due to the fact that even in the approach of the aortic valve, in which the topography of the incision is most often done in the midline and through full or partial sternotomy, the literature reports conflicting results when comparing postoperative pain between the two approaches.

### Limitations

In addition to the limitations already mentioned, the absence of a double-blinded study that met the inclusion criteria to be included in this meta-analysis is relevant, due to the fact that Colditz et al.^[27]^ demonstrated that clinical trials, with or without randomization, that do not use a double-blind design are more likely to show an innovation advantage over standard treatment. However, double blinding is challenging in comparing surgical approaches, since the surgeon is always aware of the approach used. This also contributed to the majority of studies being classified as low quality, using the Jadad scale^[5]^. Thus, to enhance the robustness of future meta-analyses, new studies may also include blinding evaluators, as suggested by Kirmani et al.^[22]^.

Given the importance of the subject, an adjuvant strategy that has recently been explored for the management of postoperative pain in cardiac surgery is blocks with locoregional anesthesia. In median sternotomy, RCTs have demonstrated a reduction in pain scores and/or the need for postoperative analgesics in adult^[28-32]^ and pediatric^[33-35]^ patients who underwent locoregional blocks. Regarding MICS, other RCTs showed conflicting results^[36-38]^. More studies are needed to better elucidate the efficacy and identify which blockade is most appropriate, depending on the type of approach in cardiac surgery.

We do not include studies in cardiac surgery involving robotic systems, because the articles on this topic did not fill the inclusion criteria of this review.

## CONCLUSION

Given the current evidence, we cannot state that FS is a more painful approach to cardiac surgery. AVR demonstrates lower pain levels using MICS techniques in the first days, but when the estimation was made by the mean of the measurements on the days when postoperative pain was evaluated, there is no difference between the groups. For MVS, the results are limited due to the methodological difference between the studies. In CABG, postoperative pain levels are equal between FS and MICS when LITA harvest was performed under direct vision. It seems that postoperative pain is more closely linked to the degree of retraction and pressure of the retractions on the tissues than to the size of the incision. We noticed the lack of high-quality studies that address this topic, therefore, the future implementation of well-conducted and higher-quality clinical studies will be fundamental to reducing the gray area that is still present in this field.
